# Commentary: Estimates of Global, Regional, and National Morbidity, Mortality, and Aetiologies of Diarrhoeal Diseases: A Systematic Analysis for the Global Burden of Disease Study 2015

**DOI:** 10.3389/fmed.2018.00011

**Published:** 2018-01-29

**Authors:** Amir Abdoli, Nahid Maspi

**Affiliations:** ^1^Department of Parasitology, Faculty of Medical Sciences, Tarbiat Modares University, Tehran, Iran; ^2^Department of Parasitology, Faculty of Medical Sciences, Ilam University of Medical Sciences, Ilam, Iran

**Keywords:** systematic analysis, global burden of disease, diarrheal diseases, helminthiasis, coinfections, malnutrition

The Global Burden of Diarrheal Disease Study 2015 (GBD 2015) estimated that diarrhea was a leading cause of death and disability-adjusted life-years, especially in young children (1). Accordingly, rotavirus, *Shigella* spp and *Salmonella* spp were the leading cause of diarrhea deaths in all ages, and in children under 5 years old, rotavirus, *Cryptosporidium* spp., and *Shigella* spp. were the most causes of death (1). GBD 2015 suggested that childhood malnutrition, unsafe water, and sanitation are leading risk factors for diarrhea, particularly in sub-Saharan Africa and south Asia (1). However, the high burden of diarrheal diseases might be involved by the high burden of helminth infections, which are endemic in the same regions (2). On the one hand, helminth infections induce immune regulation toward T helper 2 and anti-inflammatory responses (3, 4). On the other hand, a potent Th1 immunity response and their inflammatory mediators are needed to defenses again microbial pathogens (3, 4). Indeed, intestinal helminths can regulate epithelial barrier function and increase epithelial permeability (5), which consequently accelerate invasion of pathogens to the intestinal barriers. Hence, helminth coinfections suppress immune responses against microbial pathogens and increase susceptibility and severity of infectious diseases, including HIV, malaria, and tuberculosis, which are the major causes of morbidity and mortality in sub-Saharan Africa and south Asia (6, 7). Helminth coinfections also increase colonization and intensity of intestinal pathogens (8, 9). In this regard, Harris et al. (10) observed that *Vibrio cholerae* infected patients who coinfected with helminths had lower antibody response to cholera toxins. In the case of *Salmonella* and schistosomiasis coinfections, both organisms synergically induce immunological alterations that lead to increased disease duration of both infections. Importantly, *Schistosomia* can act as a latent carrier of *Salmonella*, and *Salmonella* is able to persist in the helminth’s tegument and protected from antibacterial drugs (8). In murine models, Su et al. (11) found that coinfections of *Heligmosomoides polygyrus* and *Salmonella* Typhimurium led to poor control of bacterial replication and increased intestinal inflammation in coinfected animals than animals infected with *S*. Typhimurium alone. Su et al. (11) also reported that coinfected animals had reduce in the recruitment of neutrophils and reduced in expression of chemoattractants CXCL2/macrophage inflammatory protein 2 and CXCL1/keratinocyte-derived chemokine in the site of *Salmonella* infection (11). Bobat et al. (12) demonstrated that coinfection with *Nippostrongylus brasiliensis* decrease protective immunity during natural infection or immunization with *Salmonella* Typhimurium in a murine model. Reynolds et al. (9) found that *H. polygyrus* coinfection enhance colonization and virulence of *Salmonella* by disruption of intestinal metabolome. Coinfection with *H. polygyrus* also impaired autophagy-mediated property of macrophages to killing the enteropathogen *Citrobacter rodentium* in a mouse model (13). Bednarska et al. (14) investigated the interaction of *Heligmosomoides bakeri* and *Cryptosporidium parvum* coinfection in C57BL/6 mice. They found that helminth coinfection led to prolonged course and intensity of *C. parvum* infection (14). Reese et al. (15) found that helminth infection reactivated latent γ-herpesvirus infection in a murine model *via* induction of anti-inflammatory cytokine interleukin-4 (IL-4) and the activation of the transcription factor STAT6. Reese et al. also demonstrated that IL-4 blocked the antiviral effects of interferon-γ and promoted viral replication (15). Osborne et al. (16) found that concurrent of helminths with murine norovirus infection impaired antiviral immunity alongside with changes in the intestinal microbiota. Interestingly, they observed that helminth induce immunomodulation and impaired antiviral immunity even in germ-free mice, which indicated a microbiota-independent mechanism of immunomodulation (16). Taken together, helminth coinfections can augment diarrheal diseases *via* increased susceptibility and severity of intestinal pathogens.

GBD 2015 also discussed that, “malnutrition or regular illness during the first few years of life has negative effects on future cognitive development, education, and productivity”. It is well documented that malnutrition is an important cause of immune suppression, which consequently enhance susceptibility and severity to intestinal pathogens (17). On the other hand, chronic helminth infections are linked to different insidious persistent health conditions such as protein-calorie undernutrition, anemia, growth stunting, and fatigue (3). Chronic helminth infections are also linked to poor cognitive development in children (3, 18). Thus, helminth infections could have indirect negative effects on future cognitive development, education, and productivity of children.

In conclusion, helminth coinfections might be a neglected risk factor for control of diarrheal diseases due to intestinal pathogens, so control and treatment of helminths may help to control diarrheal diseases due to microbial pathogens. Although, randomized trials are needed to demonstrate the effects of helminth coinfections on diarrheal diseases.

## Author Contributions

AA and NM conceived and designed the manuscript. AA wrote the manuscript.

## Conflict of Interest Statement

The authors declare that the research was conducted in the absence of any commercial or financial relationships that could be construed as a potential conflict of interest. The reviewer MC and handling editor declared their shared affiliation.
